# Bis[*O*-propyl (4-eth­oxy­phen­yl)dithio­phospho­nato-κ^2^ *S*,*S*′]nickel(II)

**DOI:** 10.1107/S1600536812047368

**Published:** 2012-11-24

**Authors:** Shirveen Sewpersad, Bernard Omondi, Werner E. Van Zyl

**Affiliations:** a School of Chemistry and Physics, University of KwaZulu-Natal, Westville Campus, Private Bag X54001, Durban 4000, South Africa

## Abstract

The title compound, [Ni(C_11_H_16_O_2_PS_2_)_2_], contains a four-coordinate Ni^II^ cation with an idealized square-planar geometry. The metal atom is surrounded by two chelating isobidentate dithio­phospho­nate ligands in a *trans* or *anti* configuration, binding through the S-donor atoms.

## Related literature

For information on the first structure of an Ni^II^–dithiophosphonate complex, see: Hartung (1967 ▶). For general preparative procedures for dithiophosphonates, see: Van Zyl (2010 ▶); Van Zyl & Fackler (2000 ▶). For a comprehensive review on dithiophosphonates, see: Van Zyl & Woollins (2012 ▶). For reports on the synthesis and structures of different types of Ni^II^–dithiophosphonate complexes, see: Liu *et al.* (2004 ▶); Gray *et al.* (2004 ▶); Aragoni *et al.* (2007 ▶); Arca *et al.* (1997 ▶). 
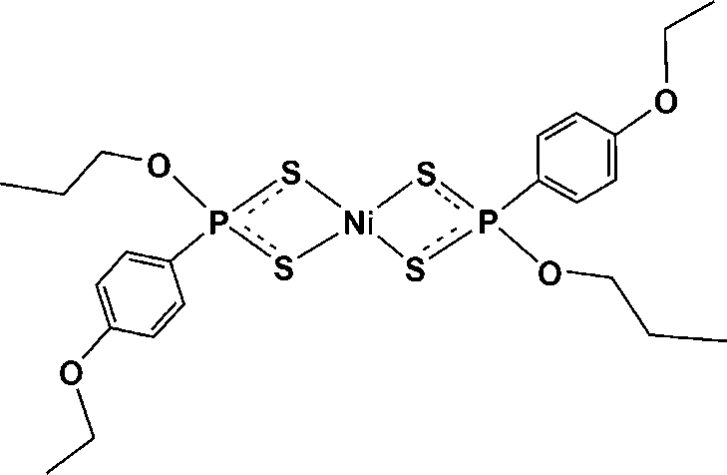


## Experimental

### 

#### Crystal data

[Ni(C_11_H_16_O_2_PS_2_)_2_]
*M* *_r_* = 609.37
Monoclinic, 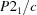

*a* = 9.4227 (2) Å
*b* = 15.6479 (3) Å
*c* = 9.5281 (2) Å
β = 102.878 (1)°
*V* = 1369.54 (5) Å^3^
*Z* = 2
Mo *K*α radiation
μ = 1.16 mm^−1^
*T* = 173 K
0.40 × 0.34 × 0.11 mm

#### Data collection

Bruker APEXII CCD diffractometer
Absorption correction: multi-scan (*SADABS*; Bruker, 2008 ▶) *T*_min_ = 0.655, *T*_max_ = 0.883
32798 measured reflections
3446 independent reflections
3335 reflections with *I* > 2σ(*I*)
*R*_int_ = 0.020

#### Refinement

*R*[*F* ^2^ > 2σ(*F* ^2^)] = 0.019
*wR*(*F* ^2^) = 0.053
*S* = 1.09
3446 reflections
153 parameters
H-atom parameters constrained
Δρ_max_ = 0.35 e Å^−3^
Δρ_min_ = −0.39 e Å^−3^

### 

Data collection: *APEX2* (Bruker, 2008 ▶); cell refinement: *SAINT-Plus* (Bruker, 2008 ▶); data reduction: *SAINT-Plus* and *XPREP* (Bruker, 2008 ▶); program(s) used to solve structure: *SHELXS97* (Sheldrick, 2008 ▶); program(s) used to refine structure: *SHELXL97* (Sheldrick, 2008 ▶); molecular graphics: *SHELXTL* (Sheldrick, 2008 ▶); software used to prepare material for publication: *SHELXL97*.

## Supplementary Material

Crystal structure: contains datablock(s) I, global. DOI: 10.1107/S1600536812047368/fj2606sup1.cif

Structure factors: contains datablock(s) I. DOI: 10.1107/S1600536812047368/fj2606Isup2.hkl

Additional supplementary materials:  crystallographic information; 3D view; checkCIF report

## Figures and Tables

**Fig. 1.**
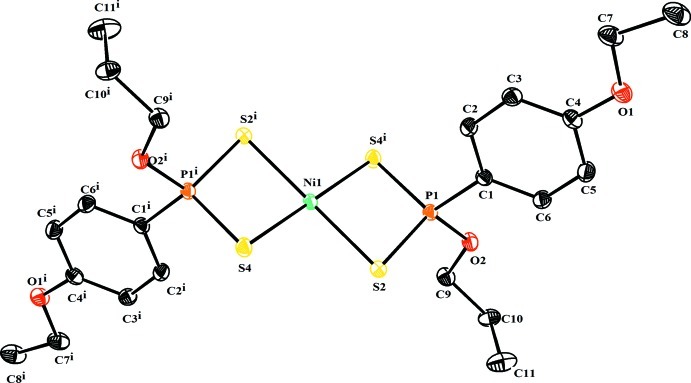
The ORTEP molecular structure of the title complex, shown with 50% probability.

## supplementary crystallographic information

### Comment

The phosphor-1,1,-dithiolate class of compounds is the heavier and softer congener of the more popular phosphonate derivatives (Van Zyl, 2010). It contains the S_2_P functionality as a common feature and several sub-categories are known which include the dithiophosphato [S_2_P(OR)_2_]¯, (*R* = typically alkyl), dithiophosphinato [S_2_P*R*_2_]¯ (*R* = alkyl or aryl), and dithiophosphonato [S_2_PR(OR')]¯, (*R* = typically aryl or ferrocenyl, *R*' = alkyl) monoanionic ligands. The latter may be described as a hybrid of the former two, and are also much less developed.

Amongst all metals involved in the coordination chemistry of dithiophosphonato ligands, however, nickel(II) is by far the best represented [Liu *et al.* (2004); Gray *et al.* (2004); Aragoni *et al.* (2007); Arca *et al.* (1997); Van Zyl & Woollins, (2012)] with the first X-ray structural report of a nickel(II) dithiophosphonate complex reported more than 4 decades ago (Hartung, 1967). The structure of the title complex does not differ significantly from related Ni^II^ complexes previously reported (see related literature). The Ni—S bond length is 2.2254 (2) and 2.2264 (2) Å, which is an insignificantly small difference to be considered anisobidentate. The Ni—P bond length is 2.8310 (3) Å, and the S—P bond length is 2.0081 (3) and 2.0026 (4) Å, respectively.

The complex in the present study was formed from the reaction between NiCl_2_.6H_2_0 and the ammonium salt of [S_2_P(OPr)(4-C_6_H_4_OEt)] (molar ratio 1:2) in an aqueous/methanolic solution, the NH_4_Cl by-product was dissolved and the precipitated product filtered off and washed with water. General and convenient methods to prepare dithiophosphonate salt derivatives have been reported (Van Zyl & Fackler, 2000).

### Experimental

A colorless methanol (40 ml) solution of NH_4_[S_2_P(OPr)(4-C_6_H_4_OEt)] (997 mg, 3.398 mmol) was prepared. A second green solution of NiCl_2_.6H_2_0 (424 mg, 1.699 mmol) in deionized water (20 ml) was prepared, and added to the colorless solution with stirring over a period of 5 min. This resulted in a purple precipitate indicating the formation of the title complex. The precipitate was collected by vacuum filtration, washed with water (3 *x* 10 ml) and allowed to dry under vacuum for a period of 3 hrs, yielding a dry, free-flowing purple powder. Purple crystals suitable for X-ray analysis were grown by the slow diffusion of hexane into a dichloromethane solution of the title complex. Yield: 740 mg, 30%. *M*.p. 122°C.

^31^P NMR (CDCl_3_): δ (p.p.m.): 101.27. ^1^H NMR (CDCl_3_): δ (p.p.m.): 7.95 (2*H*, dd, J(^31^P-^1^H) = 13.96 Hz, J(^1^H -^1^H) = 8.80 Hz, *o*-ArH), 6.94 (2*H*, dd, J(^31^P-^1^H) = 8.82 Hz, J(^1^H -^1^H) =3.06 Hz, *m*-ArH), 4.26 (2*H*, dt, J(^1^H -^1^H) = 7.89 Hz, POCH_2_), 4.06 (2*H*, q, J(^1^H-^1^H) = 6.97 Hz, ArOCH_2_), 1.74 (2*H*, m, J(^1^H -^1^H) = 7.16 Hz, POCH_2_CH_2_), 1.41 (3*H*, t, J(^1^H -^1^H) = 6.98 Hz, ArOCH_2_CH_3_), 0.96 (3*H*, t, J(^1^H -^1^H) = 7.38 Hz, POCH_2_CH_2_CH_3_).

### Refinement

All hydrogen atoms were found in the difference electron density maps and were placed in idealized positions and refined with geometrical constraints, with C—H bond lengths in the range 0.95–1.00 Å. The structure was refined to *R* factor of 0.0193.

### Crystal data

**Table:**

[Ni(C_11_H_16_O_2_PS_2_)_2_]	*F*(000) = 636
*M**_r_* = 609.37	*D*_x_ = 1.478 Mg m^−^^3^
Monoclinic, *P*2_1_/*c*	Mo *K*α radiation, λ = 0.71073 Å
Hall symbol: -P 2ybc	Cell parameters from 32798 reflections
*a* = 9.4227 (2) Å	θ = 2.2–28.8°
*b* = 15.6479 (3) Å	µ = 1.16 mm^−^^1^
*c* = 9.5281 (2) Å	*T* = 173 K
β = 102.878 (1)°	Block, purple
*V* = 1369.54 (5) Å^3^	0.40 × 0.34 × 0.11 mm
*Z* = 2	

### Data collection

**Table:**

Bruker APEXII CCD diffractometer	3446 independent reflections
Radiation source: fine-focus sealed tube	3335 reflections with *I* > 2σ(*I*)
Graphite monochromator	*R*_int_ = 0.020
φ and ω scans	θ_max_ = 28.5°, θ_min_ = 2.2°
Absorption correction: multi-scan (*SADABS*; Bruker, 2008)	*h* = −12→12
*T*_min_ = 0.655, *T*_max_ = 0.883	*k* = −20→20
32798 measured reflections	*l* = −12→12

### Refinement

**Table:**

Refinement on *F*^2^	Primary atom site location: structure-invariant direct methods
Least-squares matrix: full	Secondary atom site location: difference Fourier map
*R*[*F*^2^ > 2σ(*F*^2^)] = 0.019	Hydrogen site location: inferred from neighbouring sites
*wR*(*F*^2^) = 0.053	H-atom parameters constrained
*S* = 1.09	*w* = 1/[σ^2^(*F*_o_^2^) + (0.0288*P*)^2^ + 0.5042*P*] where *P* = (*F*_o_^2^ + 2*F*_c_^2^)/3
3446 reflections	(Δ/σ)_max_ = 0.001
153 parameters	Δρ_max_ = 0.35 e Å^−^^3^
0 restraints	Δρ_min_ = −0.39 e Å^−^^3^

### Special details

**Table:**

Geometry. All e.s.d.'s (except the e.s.d. in the dihedral angle between two l.s. planes) are estimated using the full covariance matrix. The cell e.s.d.'s are taken into account individually in the estimation of e.s.d.'s in distances, angles and torsion angles; correlations between e.s.d.'s in cell parameters are only used when they are defined by crystal symmetry. An approximate (isotropic) treatment of cell e.s.d.'s is used for estimating e.s.d.'s involving l.s. planes.
Refinement. Refinement of *F*^2^ against ALL reflections. The weighted *R*-factor *wR* and goodness of fit *S* are based on *F*^2^, conventional *R*-factors *R* are based on *F*, with *F* set to zero for negative *F*^2^. The threshold expression of *F*^2^ > σ(*F*^2^) is used only for calculating *R*-factors(gt) *etc*. and is not relevant to the choice of reflections for refinement. *R*-factors based on *F*^2^ are statistically about twice as large as those based on *F*, and *R*- factors based on ALL data will be even larger.

### Fractional atomic coordinates and isotropic or equivalent isotropic displacement parameters (Å^2^)

**Table:**

	*x*	*y*	*z*	*U*_iso_*/*U*_eq_	
C1	0.29971 (11)	−0.07749 (6)	0.10196 (10)	0.01471 (18)	
C2	0.31929 (11)	−0.15981 (7)	0.15941 (11)	0.01713 (19)	
H2	0.3857	−0.1688	0.2490	0.021*	
C3	0.24319 (11)	−0.22889 (7)	0.08761 (11)	0.01787 (19)	
H3	0.2576	−0.2847	0.1275	0.021*	
C4	0.14523 (11)	−0.21525 (7)	−0.04391 (11)	0.01670 (19)	
C5	0.12225 (11)	−0.13257 (7)	−0.10066 (11)	0.0193 (2)	
H5	0.0537	−0.1233	−0.1889	0.023*	
C6	0.19899 (11)	−0.06433 (7)	−0.02877 (11)	0.01805 (19)	
H6	0.1835	−0.0084	−0.0680	0.022*	
C7	0.08205 (13)	−0.36349 (7)	−0.07133 (13)	0.0237 (2)	
H7A	0.0514	−0.3672	0.0214	0.028*	
H7B	0.1845	−0.3826	−0.0560	0.028*	
C8	−0.01499 (13)	−0.41831 (8)	−0.18383 (15)	0.0293 (3)	
H8A	−0.1153	−0.3973	−0.2004	0.044*	
H8B	−0.0113	−0.4776	−0.1501	0.044*	
H8C	0.0187	−0.4156	−0.2739	0.044*	
C9	0.45560 (11)	0.16125 (6)	0.09836 (11)	0.01833 (19)	
H9A	0.5607	0.1608	0.0983	0.022*	
H9B	0.4439	0.1817	0.1935	0.022*	
C10	0.37431 (12)	0.21896 (7)	−0.01933 (13)	0.0217 (2)	
H10A	0.3759	0.1934	−0.1140	0.026*	
H10B	0.4247	0.2748	−0.0132	0.026*	
C11	0.21705 (14)	0.23324 (9)	−0.00963 (17)	0.0339 (3)	
H11A	0.1684	0.1779	−0.0093	0.051*	
H11B	0.1666	0.2668	−0.0927	0.051*	
H11C	0.2149	0.2642	0.0793	0.051*	
O1	0.06717 (8)	−0.27749 (5)	−0.12533 (8)	0.02031 (15)	
O2	0.39444 (8)	0.07533 (5)	0.06998 (8)	0.01738 (15)	
P1	0.39744 (3)	0.010268 (16)	0.19771 (3)	0.01352 (6)	
S2	0.30614 (3)	0.057093 (16)	0.35296 (3)	0.01596 (6)	
S4	0.40325 (3)	0.020730 (18)	0.69008 (3)	0.01702 (6)	
Ni1	0.5000	0.0000	0.5000	0.01246 (5)	

### Atomic displacement parameters (Å^2^)

**Table:**

	*U*^11^	*U*^22^	*U*^33^	*U*^12^	*U*^13^	*U*^23^
C1	0.0179 (4)	0.0127 (4)	0.0136 (4)	−0.0006 (3)	0.0037 (3)	−0.0009 (3)
C2	0.0203 (4)	0.0160 (5)	0.0145 (4)	0.0006 (4)	0.0025 (3)	0.0016 (4)
C3	0.0214 (5)	0.0135 (4)	0.0188 (5)	−0.0003 (4)	0.0049 (4)	0.0014 (4)
C4	0.0166 (4)	0.0159 (5)	0.0185 (4)	−0.0024 (4)	0.0058 (4)	−0.0034 (4)
C5	0.0198 (5)	0.0195 (5)	0.0167 (5)	−0.0009 (4)	−0.0003 (4)	0.0002 (4)
C6	0.0218 (5)	0.0142 (4)	0.0169 (5)	0.0003 (4)	0.0016 (4)	0.0017 (3)
C7	0.0246 (5)	0.0148 (5)	0.0315 (6)	−0.0027 (4)	0.0060 (4)	−0.0044 (4)
C8	0.0228 (5)	0.0204 (5)	0.0436 (7)	−0.0046 (4)	0.0050 (5)	−0.0106 (5)
C9	0.0231 (5)	0.0132 (4)	0.0191 (5)	−0.0042 (4)	0.0054 (4)	−0.0005 (4)
C10	0.0220 (5)	0.0158 (5)	0.0283 (5)	0.0011 (4)	0.0079 (4)	0.0049 (4)
C11	0.0239 (6)	0.0256 (6)	0.0537 (8)	0.0051 (5)	0.0119 (5)	0.0057 (6)
O1	0.0216 (4)	0.0159 (3)	0.0225 (4)	−0.0040 (3)	0.0029 (3)	−0.0041 (3)
O2	0.0258 (4)	0.0121 (3)	0.0136 (3)	−0.0032 (3)	0.0030 (3)	0.0007 (3)
P1	0.01689 (12)	0.01224 (12)	0.01123 (12)	−0.00026 (8)	0.00272 (9)	0.00008 (8)
S2	0.01740 (12)	0.01656 (12)	0.01382 (11)	0.00237 (8)	0.00325 (9)	−0.00129 (8)
S4	0.01565 (11)	0.02265 (13)	0.01304 (12)	0.00131 (9)	0.00382 (9)	−0.00118 (9)
Ni1	0.01398 (9)	0.01286 (9)	0.01053 (9)	−0.00043 (6)	0.00272 (7)	−0.00086 (6)

### Geometric parameters (Å, º)

**Table:**

C1—C2	1.3957 (14)	C9—C10	1.5089 (14)
C1—C6	1.4028 (13)	C9—H9A	0.9900
C1—P1	1.7865 (10)	C9—H9B	0.9900
C2—C3	1.3899 (14)	C10—C11	1.5216 (16)
C2—H2	0.9500	C10—H10A	0.9900
C3—C4	1.3973 (14)	C10—H10B	0.9900
C3—H3	0.9500	C11—H11A	0.9800
C4—O1	1.3554 (12)	C11—H11B	0.9800
C4—C5	1.4004 (14)	C11—H11C	0.9800
C5—C6	1.3825 (14)	O2—P1	1.5822 (7)
C5—H5	0.9500	P1—S4^i^	2.0026 (4)
C6—H6	0.9500	P1—S2	2.0081 (3)
C7—O1	1.4364 (13)	P1—Ni1	2.8310 (3)
C7—C8	1.5113 (16)	S2—Ni1	2.2264 (2)
C7—H7A	0.9900	S4—P1^i^	2.0026 (4)
C7—H7B	0.9900	S4—Ni1	2.2254 (2)
C8—H8A	0.9800	Ni1—S4^i^	2.2254 (2)
C8—H8B	0.9800	Ni1—S2^i^	2.2264 (2)
C8—H8C	0.9800	Ni1—P1^i^	2.8310 (3)
C9—O2	1.4640 (12)		
			
C2—C1—C6	119.20 (9)	C9—C10—H10B	109.1
C2—C1—P1	120.07 (7)	C11—C10—H10B	109.1
C6—C1—P1	120.69 (8)	H10A—C10—H10B	107.8
C3—C2—C1	121.07 (9)	C10—C11—H11A	109.5
C3—C2—H2	119.5	C10—C11—H11B	109.5
C1—C2—H2	119.5	H11A—C11—H11B	109.5
C2—C3—C4	119.20 (9)	C10—C11—H11C	109.5
C2—C3—H3	120.4	H11A—C11—H11C	109.5
C4—C3—H3	120.4	H11B—C11—H11C	109.5
O1—C4—C3	124.71 (9)	C4—O1—C7	118.10 (8)
O1—C4—C5	115.18 (9)	C9—O2—P1	120.67 (6)
C3—C4—C5	120.12 (9)	O2—P1—C1	100.55 (4)
C6—C5—C4	120.23 (9)	O2—P1—S4^i^	114.86 (3)
C6—C5—H5	119.9	C1—P1—S4^i^	113.69 (3)
C4—C5—H5	119.9	O2—P1—S2	113.25 (3)
C5—C6—C1	120.15 (9)	C1—P1—S2	113.57 (3)
C5—C6—H6	119.9	S4^i^—P1—S2	101.530 (15)
C1—C6—H6	119.9	O2—P1—Ni1	139.70 (3)
O1—C7—C8	106.40 (10)	C1—P1—Ni1	119.74 (3)
O1—C7—H7A	110.4	S4^i^—P1—Ni1	51.409 (9)
C8—C7—H7A	110.4	S2—P1—Ni1	51.421 (9)
O1—C7—H7B	110.4	P1—S2—Ni1	83.742 (11)
C8—C7—H7B	110.4	P1^i^—S4—Ni1	83.894 (11)
H7A—C7—H7B	108.6	S4^i^—Ni1—S4	180.0
C7—C8—H8A	109.5	S4^i^—Ni1—S2^i^	91.498 (9)
C7—C8—H8B	109.5	S4—Ni1—S2^i^	88.502 (9)
H8A—C8—H8B	109.5	S4^i^—Ni1—S2	88.502 (9)
C7—C8—H8C	109.5	S4—Ni1—S2	91.498 (9)
H8A—C8—H8C	109.5	S2^i^—Ni1—S2	180.0
H8B—C8—H8C	109.5	S4^i^—Ni1—P1^i^	135.304 (8)
O2—C9—C10	107.37 (8)	S4—Ni1—P1^i^	44.696 (8)
O2—C9—H9A	110.2	S2^i^—Ni1—P1^i^	44.837 (8)
C10—C9—H9A	110.2	S2—Ni1—P1^i^	135.163 (8)
O2—C9—H9B	110.2	S4^i^—Ni1—P1	44.696 (8)
C10—C9—H9B	110.2	S4—Ni1—P1	135.304 (8)
H9A—C9—H9B	108.5	S2^i^—Ni1—P1	135.163 (8)
C9—C10—C11	112.50 (10)	S2—Ni1—P1	44.837 (8)
C9—C10—H10A	109.1	P1^i^—Ni1—P1	180.0
C11—C10—H10A	109.1		
			
C6—C1—C2—C3	1.47 (15)	C6—C1—P1—Ni1	153.56 (7)
C1—C2—C3—C4	−0.24 (15)	O2—P1—S2—Ni1	−135.98 (3)
C2—C3—C4—C5	−1.32 (15)	C1—P1—S2—Ni1	110.14 (4)
C3—C4—C5—C6	1.64 (16)	S4^i^—P1—S2—Ni1	−12.297 (14)
C4—C5—C6—C1	−0.40 (16)	P1^i^—S4—Ni1—S2^i^	10.854 (12)
C2—C1—C6—C5	−1.14 (15)	P1^i^—S4—Ni1—S2	−169.146 (12)
O2—C9—C10—C11	68.95 (12)	P1—S2—Ni1—S4^i^	10.827 (12)
C3—C4—O1—C7	2.04 (15)	P1—S2—Ni1—S4	−169.173 (12)
C10—C9—O2—P1	−151.82 (7)	O2—P1—Ni1—S4^i^	−83.72 (5)
C9—O2—P1—C1	176.20 (7)	C1—P1—Ni1—S4^i^	97.82 (4)
C9—O2—P1—S4^i^	−61.34 (8)	S2—P1—Ni1—S4^i^	−164.515 (17)
C9—O2—P1—S2	54.69 (8)	O2—P1—Ni1—S4	96.28 (5)
C9—O2—P1—Ni1	−2.44 (10)	C1—P1—Ni1—S4	−82.18 (4)
C2—C1—P1—O2	156.81 (8)	S2—P1—Ni1—S4	15.485 (17)
C6—C1—P1—O2	−25.43 (9)	O2—P1—Ni1—S2^i^	−99.21 (5)
C2—C1—P1—S4^i^	33.54 (9)	C1—P1—Ni1—S2^i^	82.33 (4)
C6—C1—P1—S4^i^	−148.71 (7)	S4^i^—P1—Ni1—S2^i^	−15.485 (17)
C2—C1—P1—S2	−81.91 (8)	O2—P1—Ni1—S2	80.79 (5)
C6—C1—P1—S2	95.85 (8)	C1—P1—Ni1—S2	−97.67 (4)
C2—C1—P1—Ni1	−24.20 (10)	S4^i^—P1—Ni1—S2	164.515 (17)

Symmetry code: (i) −*x*+1, −*y*, −*z*+1.
